# Evolution of Protein Ductility in Duplicated Genes of Plants

**DOI:** 10.3389/fpls.2018.01216

**Published:** 2018-08-20

**Authors:** Inmaculada Yruela, Bruno Contreras-Moreira, A. Keith Dunker, Karl J. Niklas

**Affiliations:** ^1^Estación Experimental de Aula Dei, Consejo Superior de Investigaciones Científicas, Zaragoza, Spain; ^2^Group of Biochemistry, Biophysics and Computational Biology, Joint Unit to CSIC, Institute for Biocomputation and Physics of Complex Systems (BIFI), University of Zaragoza, Zaragoza, Spain; ^3^Fundación Agencia Aragonesa para la Investigación y el Desarrollo (ARAID), Zaragoza, Spain; ^4^Department of Biochemistry and Molecular Biology, Center for Computational Biology and Bioinformatics, Indiana University School of Medicine, Indianapolis, IN, United States; ^5^Plant Biology Section, School of Integrative Plant Science, Cornell University, Ithaca, NY, United States

**Keywords:** IDPs, polyploidy, protein ductility, protein disorder, paralogs, genome duplication, plants

## Abstract

Previous work has shown that ductile/intrinsically disordered proteins (IDPs) and residues (IDRs) are found in all unicellular and multicellular organisms, wherein they are essential for basic cellular functions and complement the function of rigid proteins. In addition, computational studies of diverse phylogenetic lineages have revealed: (1) that protein ductility increases in concert with organismic complexity, and (2) that distributions of IDPs and IDRs along the chromosomes of plant species are non-random and correlate with variations in the rates of the genetic recombination and chromosomal rearrangement. Here, we show that approximately 50% of aligned residues in paralogs across a spectrum of algae, bryophytes, monocots, and eudicots are IDRs and that a high proportion (ca. 60%) are in disordered segments greater than 30 residues. When three types of IDRs are distinguished (i.e., identical, similar and variable IDRs) we find that species with large numbers of chromosome and endoduplicated genes exhibit paralogous sequences with a higher frequency of identical IDRs, whereas species with small chromosomes numbers exhibit paralogous sequences with a higher frequency of similar and variable IDRs. These results are interpreted to indicate that genome duplication events influence the distribution of IDRs along protein sequences and likely favor the presence of identical IDRs (compared to similar IDRs or variable IDRs). We discuss the evolutionary implications of gene duplication events in the context of ductile/disordered residues and segments, their conservation, and their effects on functionality.

## Introduction

There is wide consensus that spontaneous whole genome duplications (WGD, autopolyploidy) and interspecific hybridization (allopolyploidy), followed by post-polyploid diploidization (PPD) have contributed significantly to the evolution of the land plants and to the angiosperms in particular (Wendel, 2000; Ramsey and Schemske, 2002; Soltis and Soltis, 2009; Jackson and Chen, 2010; Mandáková and Lysak, 2018; Ren et al., 2018). In general, new species emerging from either type of polyploidy tend to exhibit improved growth vigor and adaptive resilience to adverse environments thereby conferring significant evolutionary advantages (Song and Chen, 2015). Although the reasons remain unclear, plant genomes tend to have larger numbers of duplicated genes compared with the genomes of non-photosynthetic eukaryotes, although recent reports suggest that WGD events have also been frequent in insects (Li et al., 2018). Among the angiosperms, there is evidence that major clade-wide WGD events have occurred multiple times over the past 200 Mya (Lyons et al., 2008; Renny-Byfield and Wendel, 2014; del Pozo and Ramírez-Parra, 2015; Landis et al., 2018; Ren et al., 2018) in contrast to duplication events within major vertebrate lineages (Panopoulou et al., 2003; Dehal and Boore, 2005). In addition, a whole genome triplication events (triploidization, WGT, or hexaploidization) occurred in the ancestor of the core eudicots (approximately 125 Mya) and another more recent event (between 23 and 47 Mya) occurred in *Brassica* species, which affected the evolution of many agriculturally important species (Zheng et al., 2013; Kagale et al., 2014; Parkin et al., 2014). Thus, ancient and recent autopolyploidy have profoundly influenced the evolution of the flowering plants and have contributed to improved important agronomic traits, such as grain quality, fruit shape, and flowering time (Kellogg and Bennetzen, 2004; Dubcovsky and Dvorak, 2007; Leitch and Leitch, 2008; Jackson and Chen, 2010; Panchy et al., 2016). Given the fact that PPD events are recurrent over the course of angiosperm evolution, many extant diploid genomes contain a record of ancient WGD events that can be inferred by the analysis of duplicated genes with conserved co-linearity across genomic segments (Proost et al., 2012; Ren et al., 2018).

Despite their overarching importance, the consequences of polyploidy remain poorly understood. Studies have documented rapid and dynamic changes in genomic structure and gene expression in plant polyploids, which reflect genomic and functional plasticity of duplicated genes (Dubcovsky and Dvorak, 2007; Leitch and Leitch, 2008; Jackson and Chen, 2010). However, it is uncertain as to whether individual genes or WGD have contributed equally to the evolution and functional plasticity of plant genomes (see Dehal and Boore, 2005; De Storme and Mason, 2014). This ambiguity results in part because a direct causal link between an adaptive phenotype and a specific gene duplication event are difficult to ascertain because they usually occur at different times (Lawton-Rauth, 2003).

Studies during the past two decades have provided valuable information about intrinsically disordered/ductile proteins (IDPs) and disordered regions (Xie et al., 2007; Oldfield and Dunker, 2014; van der Lee et al., 2014; Wright and Dyson, 2015; Yan et al., 2016). IDPs do not fold into well-defined three-dimensional (3D) structures and can be either entirely disordered or partially disordered, with regions spanning just a few contiguous disordered residues (<10 aa) or containing long segments (≥30 aa) of contiguously disordered residues. Numerous researchers have developed algorithms that use amino acid sequences as inputs to predict the probability to be structured or disordered for each residue as outputs (He et al., 2009; Meng et al., 2017). By applying such disorder predictors to sequences of proteins with known functions, the biological activities of IDPs can be inferred from large collections of proteins (Ward et al., 2004; Xie et al., 2007). From these and other studies it has been concluded that 25–50% of all eukaryotic proteins contain at least one long IDP region and that 33–50% of eukaryotic proteomes have IDPs regions.

At the molecular level, it is uncertain how disordered/ductile proteins evolve in the scenario of WGD and PPD events. Nevertheless, there is ample evidence that disordered residues (IDRs) confer flexibility to proteomes (Tompa, 2002; Schad et al., 2011; Yruela and Contreras-Moreira, 2012; Yruela et al., 2017). Moreover, IDPs are known (1) to have played a significant role in the evolution of multicellularity and/or cell type specification (Niklas et al., 2014, 2018; Dunker et al., 2015; Niklas and Dunker, 2016; Yruela et al., 2017), (2) to contribute to organismic plasticity by facilitating protein multifunctionalities and nucleic acid interactions through complex gene regulatory network dynamics (Dyson and Wright, 2002, 2005; Xie et al., 2007; Habchi et al., 2014; van der Lee et al., 2014; O’Shea et al., 2015; Wright and Dyson, 2015; Yruela, 2015; Covarrubias et al., 2017), and (3) to be associated with proteins involved in signaling, cellular regulation, nuclear localization, chaperone activity, and RNA, DNA, and protein binding among many other functions (Xie et al., 2007; Kovacs et al., 2008; Babu et al., 2011; Buljan et al., 2013; Pazos et al., 2013; Oldfield and Dunker, 2014; Skupien-Rabian et al., 2016; Olsen et al., 2017). Moreover, IDPs and IDRs collaborate with alternative splicing (AS) and post-transcriptional modifications (PTMs) to markedly enhance the complexity of signaling networks (Niklas et al., 2015). By means of these collaborations, the same gene products bring about alternative signaling outcomes that depend on the use by IDPs or IDRs of shape-shifts to bind to multiple, different partners and that depend on the further alteration of partner binding by AS and/or PTMs (Dunker et al., 2008, 2015; Rautureau et al., 2010; Hsu et al., 2013; Niklas et al., 2015; Zhou et al., 2018). This collaboration of IDPs or IDRs with AS and/or PTMs appears to have contributed significantly to the evolution of multicellularity in all major eukaryotic lineages (Niklas et al., 2018).

The goal of this paper is to evaluate the hypothesis that WGD (or WTG) events have disproportionately increased IDRs in plant proteomes thereby contributing to plant “evolvability.” To investigate this hypothesis, we determined the fraction of IDRs in co-linear paralogs of several model and economically important plant species including green algae, bryophytes, monocots, and eudicots.

## Materials and Methods

### Protein Sequences

Protein sequences of co-linear paralogs of one chlorophyte (*Chlamydomonas reinhardtii, n* = 32), one bryophyte (*Physcomitrella patens, n* = 3,716), four monocots (*Zea mays, n* = 14,062; *Sorghum bicolor, n* = 5,336; *Oryza sativa, n* = 6,503; *Brachypodium distachyon, n* = 4,670), and thirteen eudicots (*Glycine max, n* = 74,584; *Populus trichocarpa, n* = 27,976; *Brassica oleracea, n* = 41,318; *Manihot esculenta, n* = 18,954; *Vitis vinifera, n* = 8,836; *Gossypium raimondi, n* = 25,880; *Capsicum annuun, n* = 996; *Solanum lycopersicum, n* = 6,796; *Arabidopsis thaliana, n* = 7,894; *Prunus persica, n* = 4,784; *Beta vulgaris, n* = 774; *Medicago truncatula, n* = 6,664; *Cucumis sativus, n* = 2,198) were retrieved from PLAZA 4.0,^1^ (van Bel et al., 2017). These species were selected because (1) some are model experimental systems (e.g., *C. reinhardtii* *P. patens, A. thaliana, B. distachyon*), (2) others are economically extremely important (e.g., *Z. mays, G. max, V. vinifera*), and (3) all have full representative genome and chromosome assemblies. Co-linear regions within genomes are annotated in PLAZA 4.0 by application of the i-ADHoRe algorithm (Proost et al., 2012). Co-linear paralogs are encoded by genes from the same gene family and are located in genomic segments that share the same gene content in the same order.

### WGD and WGT in Plants

Ancient large-scale duplication (WGD) and triplication (WGT) events, or more recent duplications have been reported in the literature for the following model plant systems: *P. patens* (1 recent duplication), *Z. mays* (6 duplications), *S. bicolor* (5 duplications), *O. sativa* (5 duplications), *B. distachyon* (5 duplications), *G. max* (4 duplications and 1 triplication), *P. trichocarpa* (3 duplications and 1 triplication), *V. vinifera* (2 duplications and 1 triplication), *B. oleracea* (2 duplications), *M. esculenta* (2 duplications and 1 triplication), *G. raimondi* (2 duplications and 1 undefined event), *S. lycopersicum* (2 duplications and 2 triplications), *A. thaliana* (5 duplications), *P. persica* (2 duplications and 1 triplication), *M. truncatula* (3 duplications and 1 triplication) (Panchy et al., 2016; for more details about WGD and WGT history see Gaut et al., 2000; Blanc and Wolfe, 2004; Yu et al., 2005; Schmutz et al., 2010; Song et al., 2012; Tiley et al., 2016).

Mean reported *K*s values, which represent WGDs and the divergence of duplicate gene pairs in plant families, are as follows: Angiosperms (*K*s > 3), *Solanaceae* (*K*s = 0.60), *Fabaceae* (*K*s = 0.60), *Poaceae* (*K*s = 0.90), *Brassicaceae* (*K*s = 0.80) (Schranz et al., 2012).

### Prediction of Disordered Residues

More than 60 predictors of disorder have been developed (He et al., 2009). A comparison of predictors and their variants across 1,765 proteomes reveals considerable variation in their ability to identify IDRs (Oates et al., 2013), indicating that the reliability of the predictor used in this study had to be evaluated critically before detailed analyses were under taken. In a detailed comparison of 16 commonly used predictors, PONDR VSL2b (Peng et al., 2006) had the best overall accuracy for long disordered regions (Peng and Kurgan, 2012). Nevertheless, we also explored DISOPRED v3.1 (Jones and Cozzetto, 2015) using a selected group of model monocot and eudicot species. Our analyses showed that overall DISOPRED v3.1 provided consistent results with the predictions of PONDR VSL2b (*r^2^* = 0.60 and *r^2^* = 0.96 for predicted IDRs and disordered regions with *L* > 30 aa, respectively) (**Supplementary Figures S1**–**S3**).

### Data Analysis

Bar-plots and statistical analysis were performed with Origin Pro8.6. The coefficient of determination, *r*^2^, of standard linear regression protocols were calculated as:

$$f^{2}=1-(\{\text{RSS}/\text{df}\_\{\text{Error}\}\}/\{\text{TSS}/\text{df}\_\{\text{Error}\}\})$$

where RSS is the residual sum of square and TSS is the total sum of square (Anderson-Sprecher, 1994).

### Sequence Alignment

Pairwise alignments of co-linear paralogous sequences were determined using Clustal Omega (Sievers et al., 2011). For each aligned pair, the aligned disorder predictions were compared in order to calculate three types of IDRs: (1) identical disordered residues, where both the amino acid sequence and disorder predictions were identical (denoted hereafter as “identical IDRs”), (2) similar disordered residues, where the disorder predictions matched but the amino acid sequence varied (“similar IDRs”), and (3) variable residues where disorder predictions were not conserved (“variable IDRs”) (Yruela et al., 2017). The same three IDRs types were also computed for the subset of residues that were predicted to be disordered within long segments of at least 30 contiguous disordered residues (*L* > 30 aa). In all cases the fraction of IDRs were computed by dividing the number of aligned IDRs with the total aligned residues.

The IDR categories described previously (Yruela et al., 2017) and used here were inspired by work of Bellay et al. (2011) but differ in some details reported previously by others. Bellay et al. (2011) focused on orthologs and did not consider insertions and deletions, only sequences that could be aligned. In contrast, here we are studying paralogs. The three categories of IDRs described above for our work provided useful categories for the IDRs found in these proteins. For such proteins, we were particularly interested in examples in which disordered/ductile regions were present and absent in a given paralogous pair of proteins, and as noted above, Bellay et al. (2011), did not consider insertions or deletions at all.

### Gene Ontology (GO) Enrichment Analysis

Gene Ontology annotations for the complete proteomes analyzed were retrieved from PLAZA 4.0 and genome GO term (expected) frequencies computed for each species. In addition, the subset of GO annotations corresponding to pairs of paralogs harboring long disordered segments (*L* > 30 aa) were used to compute (1) observed GO term frequencies for co-linear genes and (2) observed GO term frequencies for co-linear genes harboring at least two thirds of identical IDRs. Enrichment was computed by applying Fisher’s exact test with Bonferroni correction^2^ to compare the observed and expected GO term frequencies. When possible, plant-specific GO-slim terms were assigned to enriched terms by parsing file^3^.

## Results and Discussion

We focused on the paralogs of 19 important plant species across a broad spectrum of the Chlorobionta (i.e., green algae to eudicots) whose proteome size, basic haploid chromosome number and number of co-linear paralogous pairs differ significantly (**Table 1**). From 37 to 52% total aligned paralogous sequences were identified by PONDR VSL2b as having an IDR signature. These results are consistent with previous whole proteome analyses (Yruela and Contreras-Moreira, 2012). The highest percentages of aligned IDRs were found in monocot species (50–52%); the lowest percentage was found in the green alga *C. reinhardtii* (37%). The range of total aligned IDRs observed for the 19 species examined in this study accords reasonably well with the evolutionary origins of these taxa (i.e., total aligned IDRs tend to increase with more recent descent). It is worth nothing that these values were on average much lower than those predicted using DisoPred v3.1 (**Supplementary Table S1**) and also much lower than those previously reported (Yruela and Contreras-Moreira, 2012) using DisoPred v2.42 (Ward et al., 2004). The differences observed using both versions of DisoPred are attributed to different sensitivities to IDRs longer than 20 amino acids (Jones and Cozzetto, 2015).

**Table 1:** Characteristics of the nineteen plant species examined in this study.

Plant species	Proteome size	Haploid chromosome number (*n*)	Pair of co-linear paralogs	Fraction of total aligned IDRs PONDR VSL2	Fraction of aligned IDRs in segments *L* > 30 aa PONDR VSL2b
Chlorophyte (*n*)					
*Chlamydomonas reinhardtii*	14,488	17	16	0.37	0.55

Bryophyte					
*Physcomitrella patens*	48,022	27	1,858	0.50	0.62

Monocots (2*n* = 2×)					
*Zea mays*	58,615	10	7,031	0.52	0.66
*Sorghum bicolor*	39,248	10	2,668	0.52	0.65
*Oryza sativa*	40,881	12	3,251	0.50	0.62
*Brachypodium distachyon*	33,844	5	2,335	0.50	0.63

Eudicots (2*n* = 2×)					
*Glycine max*	71,525	20	37,292	0.45	0.56
*Populus trichocarpa*	73,110	19	13,988	0.46	0.55
*Vitis vinífera*	41,208	19	1,916	0.44	0.53
*Brassica oleracea*	56,687	9	20,660	0.48	0.57
*Manihot esculenta*	43,286	18	9,477	0.47	0.58
*Gossypium raimondi*	59,057	13	12,941	0.47	0.58
*Capsicum annuum*	40,627	12	498	0.42	0.48
*Solanum lycopersicum*	36,010	12	3,399	0.46	0.55
*Arabidopsis thaliana*	48,148	5	3,947	0.47	0.59
*Prunus persica*	32,595	8	2,392	0.47	0.58
*Beta vulgaris*	32,874	9	387	0.47	0.57
*Medicago truncatula*	57,661	8	3,322	0.47	0.57
*Cucumis sativus*	25,668	7	1,099	0.49	0.59


The fraction of aligned residues in long disordered segments (*L >* 30 aa) was also calculated using PONDR VSL2b, which revealed high proportions of IDRs located in such segments *L* > 30 aa (ca. 60%) (**Table 1**). Similar results were obtained using DisoPred v3.1 (**Supplementary Table S1**). Thus, the results consistently indicated that a high proportion of IDRs reside in long ductile segments (*L* > 30 aa) in paralogous pairs.

**FIGURE 1:**
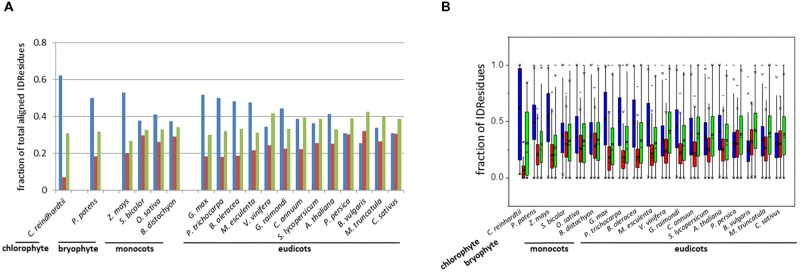
Bar-plot of the fraction **(A)** and box-plot distribution **(B)** of total aligned IDResidues. Identical IDRs (blue), similar IDRs (red), and variable IDRs (green) based on PONDR VSL2b predictions.

The distribution of the three types of IDRs (i.e., identical, similar, and variable IDRs) was analyzed for the paralogous pairs across all 19 species (**Figure 1**). Here we use the terms “identical IDRs” for those that are conserved with respect to sequence, length and location from one paralog to the next, “similar IDRs” for those that show substantial sequence variations but are conserved with respect to length and location from one paralog to the next and “variable IDRs” for those that are observed in some paralogs but absent in others.

**FIGURE 2:**
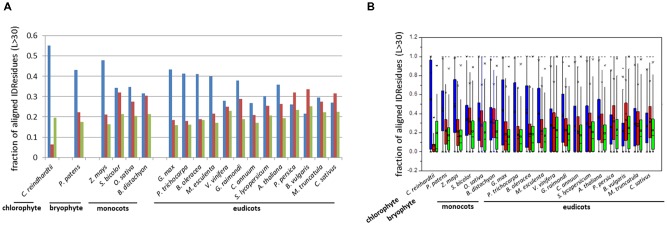
Bar-plot of the fraction **(A)** and box-plot distribution **(B)** of aligned residues in ductile regions (*L* > 30 aa). Identical IDRs (blue), similar IDRs (red), and variable IDRs (green) based on PONDR VSL2b predictions.

Analyses indicated that the percentage of aligned identical IDRs in paralogous sequences predicted by PONDR VSL2b ranged between 30 and 60%. It was highest in the green algae *C. reindhardtii* and lowest in the eudicot *B. vulgaris* (**Figure 1**). These data are in agreement with previous results (Yruela and Contreras-Moreira, 2013). The predicted fractions of similar IDRs and variable IDRs were highest in *B. vulgaris, M. truncatula, P. persica*, and *C. sativus* (**Figures 1**, **2**). We speculate that the differences observed among the three different kinds of predicted IDRs reflect the history of genome duplication/polyploidy events (i.e., both chromosome number and the number of paralogs) in the species investigated in this study (**Table 1**). It is worth noting that the basic haploid chromosome number of *B. vulgaris, M. truncatula, P. persica*, and *C. sativus* are much reduced (*n* = 7 – 9) compared with those of the green alga *C. reinhardtii* (*n* = 17), other monocots such as *O. sativa* (*n* = 12) and *Z. mays* (*n* = 10), and eudicots such as *G. max* (*n* = 20) and *P. trichocarpa* (*n* = 19) (**Table 1**). Furthermore, the combination of multiple ancestral WGD and more recent polyploidy events promoting high rates of duplicated gene retentions (e.g., *P. trichocarpa, G. max, B. oleoracea*) (Parkin et al., 2014; Panchy et al., 2016) likely also favored the increase of identical IDRs.

With the exception of the green alga *C. reinhardtii* and the bryophyte *P. patens*, a statistically significant and positive correlation (*r*^2^ = 0.45, *P* = 8 × 10^-4^) was observed between the number of co-linear paralogous pairs and the haploid number of chromosomes across the 17 vascular plant species (**Supplementary Figure S2A**). The proteome size and the number of paralogs were also significantly correlated with one another (*r*^2^ = 0.53, *P* = 5 × 10^-3^) (**Supplementary Figure S2B**).

**FIGURE 3:**
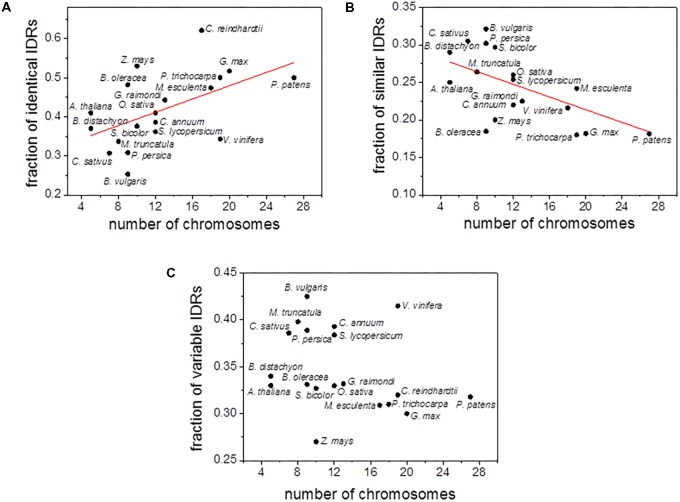
Scatter plots of chromosome numbers versus the fraction of aligned identical IDRs **(A)**, similar IDRs **(B)**, variable IDRs **(C)** for *C. reindhartii* (*n* = 17), *P. patens* (*n* = 27), *Z. mays* (*n* = 10), *S. bicolor* (*n* = 10), *O. sativa* (*n* = 12), *B. distachyon* (*n* = 5), *G. max* (*n* = 20), *P. trichocarpa* (*n* = 19), *B. oleracea* (*n* = 9), *M. esculenta* (*n* = 18), *V. vinifera* (*n* = 19), *G. raimondi* (*n* = 13), *C. annuun* (*n* = 12), *S. lycopersicum* (*n* = 12), *A. thaliana* (*n* = 5), *P. persica* (*n* = 9), *B. vulgaris* (*n* = 9), *M. truncatula* (*n* = 8), *C. sativus* (*n* = 7). Disordered predictions are based on PONDR VSL2b.

In order to further explore the relationship between polyploidy and IDRs content, we analyzed the correlation between the number of chromosomes and the fraction of the three types of IDRs (i.e., identical, similar and variable IDRs). A statistically positive and significant correlation (*r*^2^ = 0.42, *P* = 5 × 10^-3^) was observed between the number of chromosomes and the fraction of identical IDRs (**Figure 3A** and **Supplementary Figure S3A**). In contrast, a statistically significant negative correlation (*r*^2^ = 0.42, *P* = 5 × 10^-3^) was observed for the fraction of similar IDRs (**Figure 3B** and **Supplementary Figure S3B**). Little or no correlation was observed between the number of chromosomes and the fraction of variable IDRs (**Figure 3C** and **Supplementary Figure S3C**).

It has been reported that most of the retained duplicated genes in angiosperms are enriched in Gene Ontology (GO) categories associated with protein targeting, synthesis, and post-translational modification (Ren et al., 2018). In order to put in perspective our results and get additional insights, we investigated the GO annotations function of (1) co-linear paralogous proteins in all 19 plant species studied, and (2) the group of co-linear paralogous harboring a majority of identical IDRs. The analysis revealed that on average paralogs are enriched in biological processes (P) (50–60%), molecular functions (F) (20–30%) and cellular components (C) (15–30%) GO categories with corrected *p*-values < 10E-6. Similar trends were found in the group of paralogs enriched in identical IDRs (*p*-values < 10E-5). Regarding biological processes, we found that paralogs with identical IDRs are mainly associated with terms such as “catalytic activity,” “metabolic process,” “biosynthetic process,” “development,” “cell differentiation” and “cell proliferation” (*p*-values < 10E-6). The most significant association among specific molecular functions was with “molecular binding” and “transport” terms (*p*-values < 10E-6). Regarding cellular components we found that paralogs with identical IDRs are associated with “plasma membrane” and “thylakoid” terms (*p*-values < 10E-6).

Differences in the distribution of the fraction of IDRs across the co-linear paralogous sequences could be the result of differences in the locations of paralogous genes along chromosomes. This attribution is based on a positive correlation between genetic recombination rates and protein disorder frequency observation, and on the fact that ductile segments are more conserved between paralogs located in regions close to (as opposed to distant from) centromeres (Yruela and Contreras-Moreira, 2013). It is clear from previous analyses and the results presented here that significant evolutionary differences exist in proteomes and in the “dynamics” of IDRs protein sequence sorting during polyploidy events, i.e., our data indicate that polyploidy incurs a disproportionate increase in highly conserved flexibility/ductility compared with less conserved and random disordered/ductile protein regions.

Differences in the distribution of the three types of IDRs have been also observed in a set of transcription factor orthologs involved in key developmental processes such as cellular differentiation, cell division, cell cycle, and cell proliferation (Yruela et al., 2017). Analyses indicated that the fraction of predicted aligned identical IDRs is higher in the green algae (chlorophytes) and non-vascular land plants (bryophytes) compared to vascular plants and animals, whereas the fraction of less conserved IDRs (similar and variable IDRs) is lower in the green algae (chlorophytes) and the non-vascular plants in comparison to vascular plants and animals (Yruela et al., 2017).

**FIGURE 4:**
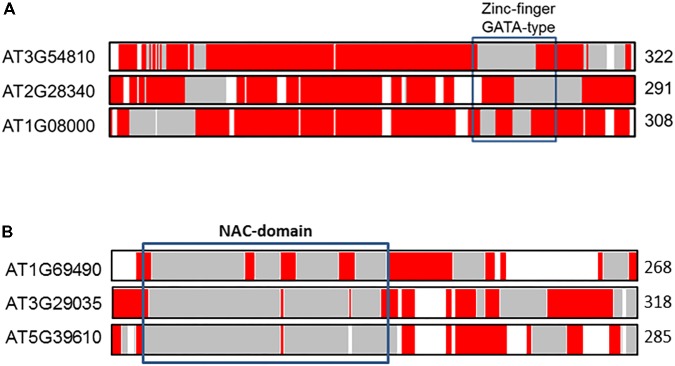
Alignments of the co-linear GATA10 **(A)** and NAC92 **(B)** paralogs in *A. thaliana*. The protein length is given to the right of each sequence. The color coded bars in each sequence depict predicted disorder. Disordered residues are shown in red, ordered residues are shown in gray, and alignment gaps are shown in white. Typical DNA-binding domains are shown as black boxes.

To illustrate differences of IDRs in paralogs compared with orthologs we selected two transcription factors of *A. thaliana* previously examined by Yruela et al. (2017). **Figure 4** shows the distribution of predicted aligned IDRs along the sequences of the GATA10 and NAC92 transcription factor paralogs, which belong to zinc finger and NAC families, respectively. Inspection of **Figure 4A** shows that co-linear paralogs of GATA10 (AT1G08000), located on chromosome 2 (AT2G28340) and chromosome 3 (AT3G54810), have important differences in the distribution of IDRs, as indicated in the marked zinc-finger GATA-type binding domain. Although the three transcription factors have a high proportion of IDRs (ca. 90%), analysis indicates that between 13 and 30% of the aligned residues correspond to identical IDRs. The percentage of similar and variable IDRs ranges from between 30 and 46%. It has been speculated that the three paralogs are involved in cell differentiation, and that they might be involved in the regulation of some light-responsive genes. We speculate further that variations observed in the distribution of IDRs around the DNA-binding motif might result in different paralog functionalities. Such differences contrast with those observed in GATA orthologs (Yruela et al., 2017). In particular the distribution of IDRs in the zinc-finger GATA-type binding domain is more conserved and manifests a progressive gain of IDRs from green algae to vascular plants, which increases flexibility/ductility in the functional domain.

The alignment of NAC92 (AT5G39610) paralogs on chromosome 3 (AT3G29035 or NAC59) and chromosome 1 (AT1G69490 or NAC29) also reveals notable differences in IDRs distributions. The percentage of total aligned IDRs is ca. 40%, and that of identical IDRs is ca. 10–20%. Such differences likely contributed to functional divergences. NAC92 and NAC59 are involved in senescence, salt stress responses, and lateral root development (Balazadeh et al., 2010), whereas NAC29 is involved in heat stress responses (Zhao et al., 2018).

**FIGURE 5:**
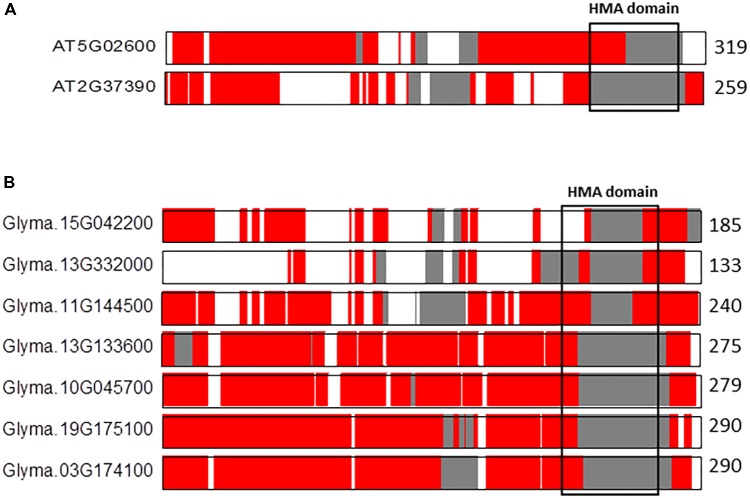
Alignments of the co-linear NAKR1 paralogs in *A. thaliana* **(A)** and *G. max* **(B)**. The protein length is given to the right of each sequence. Sequences are represented by color coded bars representing predicted disorder. Disordered residues are shown in red), ordered residues are shown in gray, and alignment gaps are shown in white. Typical DNA-binding domains are shown as black boxes.

An additional interesting example is the comparison between the co-linear NAKR paralogs in *A. thaliana* (*n* = 5) and *G. max* (*n* = 20) (**Figure 5**). The pair of co-linear NAKR1 (AT5G02600) and NAKR2 (AT2G37390) paralogs located on *A. thaliana* chromosomes 5 and 3, respectively, show notable differences in the IDRs distribution along sequences (**Figure 5A**). The percentage of total aligned IDRs is ca. 82% in contrast to the percentage of identical IDRs, which is only ca. 24%. The percentage of similar and variable IDRs is 52 and 22%, respectively. The alignment reveals once again differences in the distribution of IDRs, particularly in the functional HMA domain. NAKR1 (Sodium Potassium Root Defective1) is a heavy metal-binding protein expressed in phloem. It interacts with the FLOWERING LOCUS T (FT) transcription factor and regulates flowering through both the transcriptional regulation and transport of FT, especially in response to potassium availability (Negishi et al., 2018). The precise function of NAKR2 is still unclear. In contrast, in *G. max* the differences in the IDRs composition of the HMA domain across the seven co-linear NAKR1 paralogs are smaller, in particular among four of them, Glyma13G133600, Glyma10G045700, Glyma19G175100, and Glyma03G174100 located on *G. max* chromosomes 13, 10, 19, and 3, respectively (**Figure 5B**). The proportion of aligned IDRs on average ranges from 53 to 81%. The fraction of identical IDRs is ca. 87%, and those of similar IDRs and variable IDRs are ca. 1 and 0.6%, respectively. These observations once again support our hyphothesis that polyploidy likely favors increases in highly conserved flexibility/ductility. This fact might have preserved essential functionalities during the course of angiosperm evolution.

## Conclusion

In summary, the results reported here indicate: (1) a positive correlation between chromosome number and the fraction of paralogous sequence that are identified as identical IDRs, and (2) a negative correlation between chromosome number and the fraction of paralogous sequences that are identified as similar IDRs. We interpret these findings to indicate (1) retention of paralogs with identical IDRs after WGD (or WTG) could be favored by selection because identical IDRs (as opposed to similar/variable IDRs) facilitated essential functions involved in development, and (2) the retention of genes with high proportions of similar/variable IDRs after WGD (or WTG) could be less likely and therefore tended to be lost in one of paralogs. We argue that the patterns observed for similar/variable IDRs pattern are simply a byproduct of recent WGD (or WTG) events. Thus, ancient WGD (or WGT) events in species such as *Z. mays, G. max*, and *P. trichocarpa* have disproportionately favored an increase in aligned identical IDRs across paralogs, thereby contributing to the stability of functions such as the catalytic activity of proteins, metabolic and transport processes, and molecular binding. Based on these characteristics, it is not unreasonable to speculate that, over evolutionary time, duplication events have stabilized proteome adaptive functionalities.

## Author Contributions

IY and AKD conceived the study. IY analyzed the data, wrote the original draft of the manuscript, and reviewed and edited the manuscript. BC-M did data analyses, and reviewed and edited the manuscript. AKD and KN contributed to discussion, and wrote, reviewed, and edited the manuscript.

## Conflict of Interest Statement

The authors declare that the research was conducted in the absence of any commercial or financial relationships that could be construed as a potential conflict of interest.

## Footnotes

1 https://bioinformatics.psb.ugent.be/plaza/

2 http://stat.ethz.ch/R-manual/R-devel/library/stats/html/fisher.test.html

3 http://www.geneontology.org/ontology/subsets/goslim_plant.obo

## Supplementary Material

FIGURE S1 Average fraction **(A,B)** and box-plot distribution **(C,D)** of total aligned IDRs **(A,B)** and aligned residues in ductile regions (*L* > 30 aa). Identical IDRs (blue), similar IDRs (red), and variable IDRs (green). The data represent the average of paralogs in the proteomes of three monocots (*Z. mays, O. sativa, B. distachyon*) and two eudicots (*P. trichocarpa, A. thaliana*). Disordered predictions are based on DisoPred v3.1.

FIGURE S2 Scatter plots of **(A)** number of chromosomes versus number of paralogous pairs and **(B)** proteome size versus number of paralogous pairs in monocots (*Z. mays, O. sativa, B. distachyon*) and eudicots (*P. trichocarpa, A. thaliana*). Disordered predictions are based on DisoPred v3.1.

FIGURE S3 Scatter plots of number of chromosomes versus the fraction of aligned identical IDRs **(A)**, similar IDRs **(B)**, and variable IDRs **(C)** in monocots *Z. mays* (*n* = 10), *O. sativa* (*n* = 12), *B. distachyon* (*n* = 5), and eudicots *P. trichocarpa* (*n* = 19) and *A. thaliana* (*n* = 5). Disordered predictions are based on DisoPred v3.1.

TABLE S1 Characteristics of the plant species examined with DisoPred v3.1.
