# Supplementary figures and images for: Evolution of Protein Ductility in Duplicated Genes of Plants
Source: Front Plant Sci. 2018 Aug 20;9:1216. doi: 10.3389/fpls.2018.01216 (PMC6109787; doi:10.3389/fpls.2018.01216)

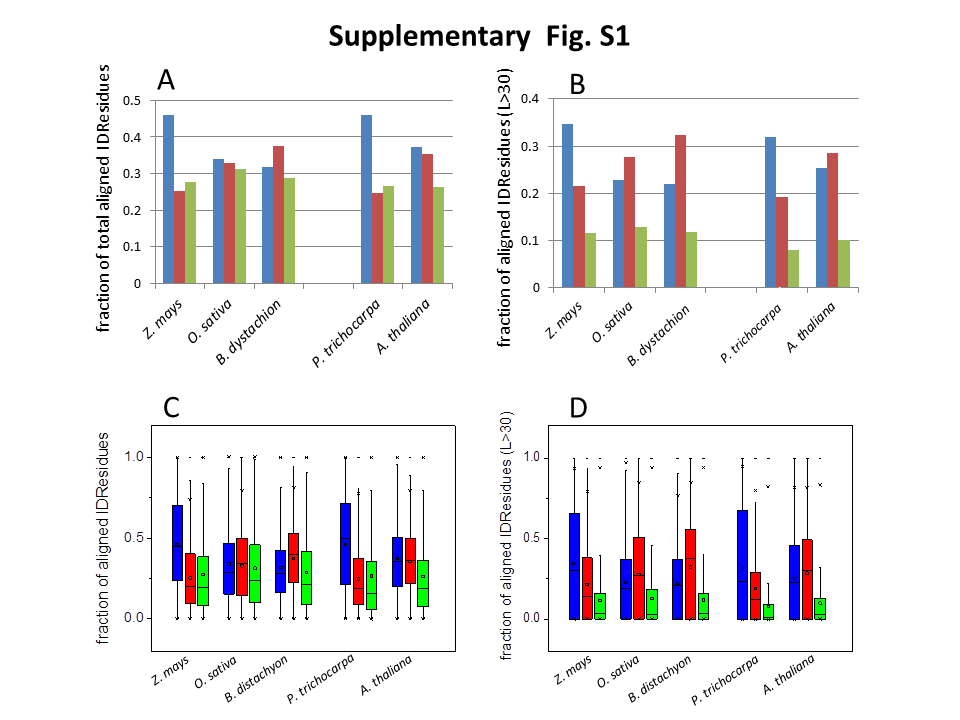

Supplement: FIGURE S1 — Average fraction (A,B) and box-plot distribution (C,D) of total aligned IDRs (A,B) and aligned residues in ductile regions (L > 30 aa). Identical IDRs (blue), similar IDRs (red), and variable IDRs (green). The data represent the average of paralogs in the proteomes of three monocots (Z. mays, O. sativa, B. distachyon) and two eudicots (P. trichocarpa, A. thaliana). Disordered predictions are based on DisoPred v3.1. [file Image_1.TIF]

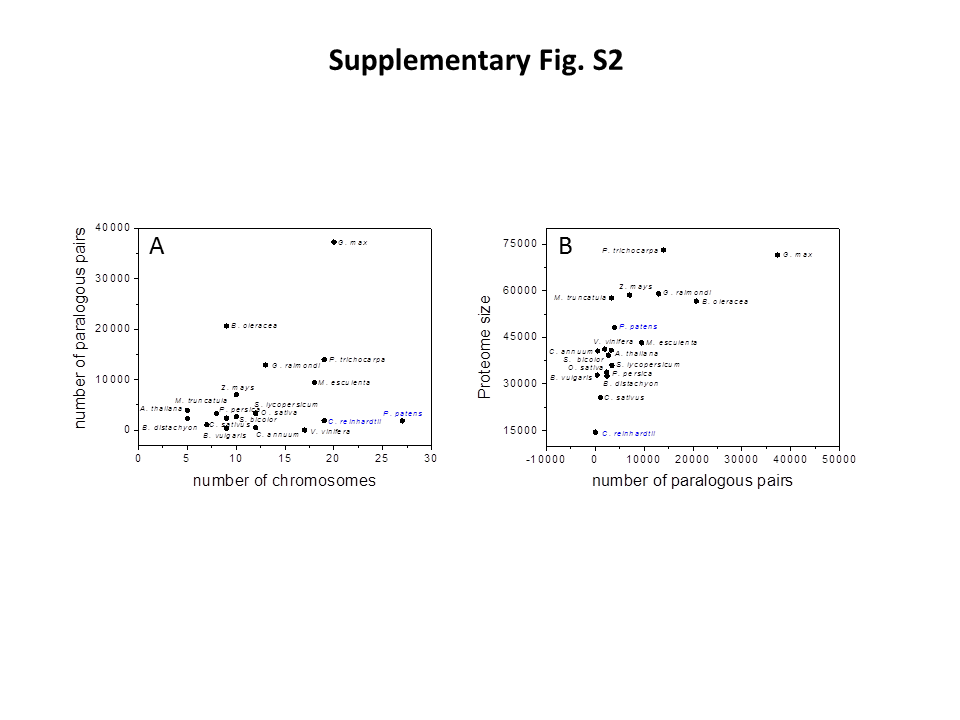

Supplement: FIGURE S2 — Scatter plots of (A) number of chromosomes versus number of paralogous pairs and (B) proteome size versus number of paralogous pairs in monocots (Z. mays, O. sativa, B. distachyon) and eudicots (P. trichocarpa, A. thaliana). Disordered predictions are based on DisoPred v3.1. [file Image_2.TIF]

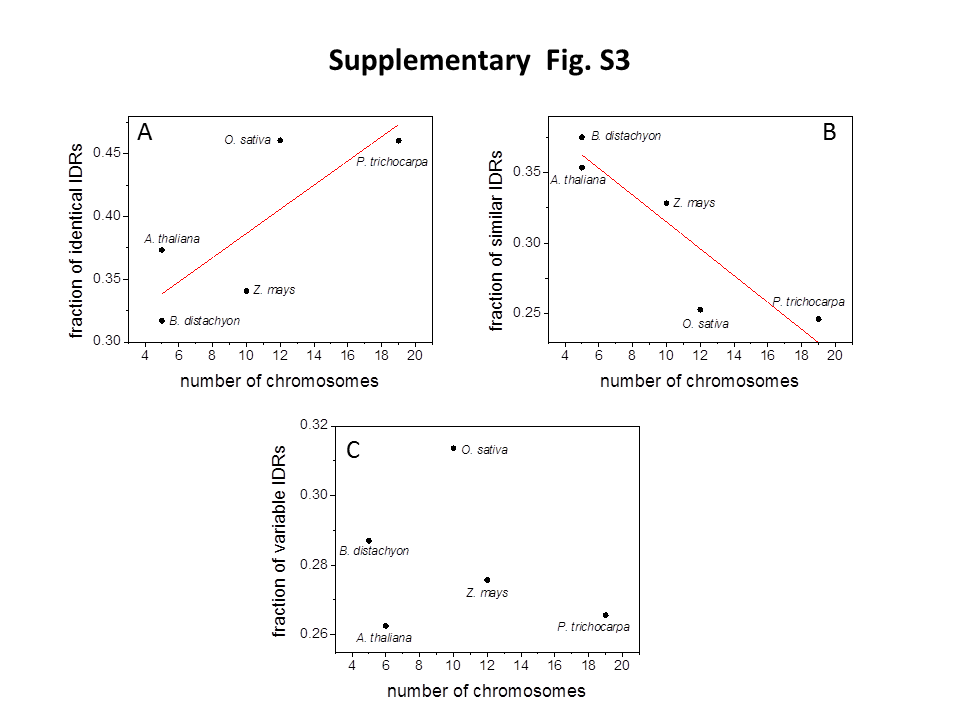

Supplement: FIGURE S3 — Scatter plots of number of chromosomes versus the fraction of aligned identical IDRs (A), similar IDRs (B), and variable IDRs (C) in monocots Z. mays (n = 10), O. sativa (n = 12), B. distachyon (n = 5), and eudicots P. trichocarpa (n = 19) and A. thaliana (n = 5). Disordered predictions are based on DisoPred v3.1. [file Image_3.TIF]
