# Supplementary material for: Evolution of Protein Ductility in Duplicated Genes of Plants
Source: Front Plant Sci. 2018 Aug 20;9:1216. doi: 10.3389/fpls.2018.01216 (PMC6109787; doi:10.3389/fpls.2018.01216)
Supplement: TABLE S1 — Characteristics of the plant species examined with DisoPred v3.1. [file Table_1.pdf]

TABLE S1. Characteristics of the plant species examined with DisoPred v3.1

Plant specie	Proteome size	Haploid chromosomes number (*n*)	Pair of co-linear paralogues	Fraction of total aligned IDRs DisoPred v3.1	Fraction of aligned IDRs in segments L>30aa DisoPred v3.1
Monocots (*2n=2x*)					
Zea mays	58,615	10	7,031	0.22	0.54
Oryza sativa	40,881	12	3,251	0.26	0.50
Brachypodium distachyon	33,844	5	2,335	0.26	0.52
Eudicots (*2n=2x*)					
Populus trichocarpa	73,110	19	13,988	0.26	0.47
Arabidopsis thaliana	48,148	6	3,947	0.25	0.50
